# An Overview of the Synthesis of Gold Nanoparticles Using Radiation Technologies

**DOI:** 10.3390/nano8110939

**Published:** 2018-11-15

**Authors:** Lucas Freitas de Freitas, Gustavo Henrique Costa Varca, Jorge Gabriel dos Santos Batista, Ademar Benévolo Lugão

**Affiliations:** Instituto de Pesquisas Energéticas e Nucleares, IPEN-CNEN/SP. Av. Prof. Lineu Prestes, No. 2242, Cidade Universitária, São Paulo 05508-000, Brazil; jorgegabriel@usp.br (J.G.d.S.B.); ablugao@gmail.com (A.B.L.)

**Keywords:** gold nanoparticle, radiolytic synthesis, radiation technologies, gamma radiation, electron beam radiation, X-ray

## Abstract

At a nano-level, optical properties of gold are unique and gave birth to an emerging platform of nanogold-based systems for diverse applications, because gold nanoparticle properties are tunable as a function of size and shape. Within the available techniques for the synthesis of gold nanoparticles, the radiolytic synthesis allows proper control of the nucleation process without the need for reducing agents, in a single step, combined or not with simultaneous sterilization. This review details and summarizes the use of radiation technologies for the synthesis and preparation of gold nanoparticles concerning fundamental aspects, mechanism, current pathways for synthesis and radiation sources, as well as briefly outlines final applications and some toxicity aspects related to nanogold-based systems.

## 1. Introduction

### 1.1. Nanomaterials

When one investigates the matter in the nanometric scale, it is possible to observe features that are different and sometimes even the opposite of those intrinsic to bulk and bigger materials. Two physical effects are known to be responsible for these unique properties from nanomaterials: one is the quantization of the electronic states, relevant to the optical and magnetic features that are significantly size-dependent, and are more apparent in nanomaterials; and the other is the higher surface to volume ratio compared to bulk materials, very important to the behavior of nanomaterials in terms of thermal, mechanical and chemical properties [1].

If they are present in suspensions, the nanomaterials tend to form colloids (by definition, a system composed of one solid or liquid phase in which a second liquid phase is in suspension). Colloids are especially attractive in research and applied science because of their intrinsic properties, such as the above-mentioned high surface to volume ratio. With a higher area of contact between the nanoparticle and the surrounding medium, the interactions are facilitated and, consequently, the ability to catalyze reactions via those interactions increases. Their optical properties, i.e., relevant extinction efficiency in the 300–1000 nm region of the light spectrum, are also suitable for biomedical and chemical protocols. The resonance of surface plasmons, defined as the collective oscillations of electrons present in the surface of metallic materials when an electromagnetic field of a specific wavelength is applied, is the main responsible for the optical properties of metallic nanomaterials, but is not the only variable that must be taken into consideration to explain other features, such as photocatalysis. Some quantum-mechanical variables other than surface plasmon resonance must be used to justify those properties [2].

Metallic nanoparticles, therefore, have gained attention as colloidal systems for a plethora of applications [3]. Relevant examples of metallic nanoparticles include iron oxide, gold, and silver, in a variety of shapes and sizes, which over the years have been extensively used, designed, and modified towards their usage in medicine and biomedical applications [4].

Although the development of monodisperse nanoparticles within the nano- to the submicron-diameter range may not be so simple to achieve, the nanotechnology potential in several areas justifies the effort from researchers towards developing different kinds of nanomaterials. Such materials may be designed for use as catalysts for both fundamental research and industry, improving reaction yields, reducing temperatures of chemical processes, and promoting enantioselectivity in asymmetric synthesis, or for biomedical applications, such as contrast agents, and so on [4,5]. In addition, when such particles are designed for biomedical applications—drug carriers, contrast imaging, theranostics, among others, due to their biological non-specificity, the nanoparticles are flexible in terms of allowing additional processes or steps for functionalization, whether by chemical or physical modification, to confer site-specific delivery, intratumor or cell uptake, modify retention times, improve drug loading, among others [5,6]. Concerning functionalization, binding, coating, or capping processes are the most common approaches, using a wide variety of ligand and biomolecules, e.g., antibodies, nucleotides, and peptides [4,6].

Radiation technology has been proved useful in nanotechnology as it has been explored mainly using high-energy charged particles, e.g., electrons and ions, as well as photons such as X-ray and gamma rays [7]. Some non-ionizing sources of radiation such as microwave and ultraviolet (UV) light under specific wavelengths may also be applied. Examples of nanostructures that may be designed and synthesized by using radiation include metal nanoparticles [8], organic nanoparticles such as proteins [9] and enzymes [10], hybrid nanoparticles [11], nanocomposites [12], nanogels [13,14], among others.

While techniques available for the synthesis of gold nanoparticles abound, radiation technologies feature relevant properties that make such technologies unique, e.g., proper control of the nucleation process depending on the dose and dose rate, no need for reducing agents and the possibility to combine nanoparticle synthesis and simultaneous sterilization. The weakness of technologies, on the other hand, relates to the low availability worldwide and restricted access to gamma irradiators, e-beam accelerators or X-ray devices if compared to conventional techniques. In addition, several materials, especially capping or stabilizing agents, may be sensitive to the high energy irradiation and thus may not work effectively. This review summarizes and details the use of low linear energy transfer (LET) radiation technologies, by means of gamma, e-beam, and X-ray, for the synthesis and preparation of gold nanoparticles, comprising fundamental aspects, the radiolytic mechanism and pathways for synthesis, the radiation source and types, and final applications of nanogold systems synthesized by the aforementioned technology.

### 1.2. Gold Nanoparticles

Noble metals such as gold, when applied for the synthesis of nanostructured materials, present all the aforementioned properties and others, such as relatively low toxicity to biological systems and conformational flexibility [15]. For millennia, colloidal gold has been empirically used for a variety of applications. The Lycurgus Cup (Roman 400 A.D.) is one of the well-known examples of the early use of colloidal gold in the manufacture of colored glass. During the recent decades, the biomedical properties of gold nanoparticles emerged, from the surface modification by the bioconjugation of specific molecules to optical features tuned for diagnostic protocols according to the morphology of the particles, among others [16]. Gold nanoparticles can assume several morphologies considering bulk gold nanospheres up to composites of a polymeric nucleus covered by a gold layer or capping. They can also be assembled in short or long chains of spherical nanoparticles with desired optical properties depending on the chain size [17].

**Figure 1:**
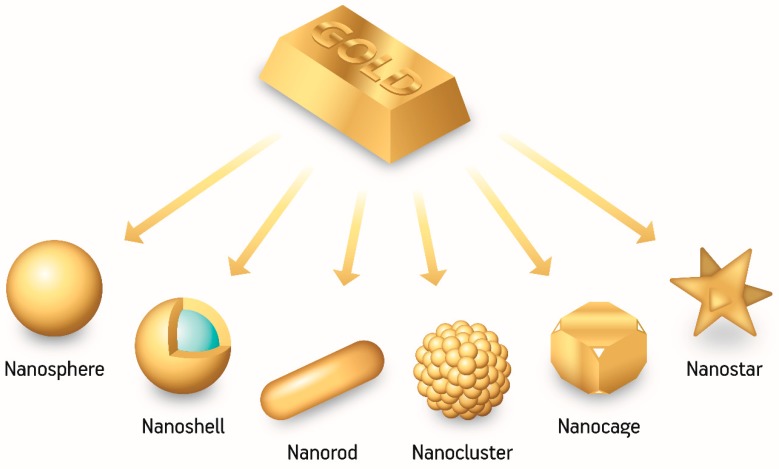
Representative scheme of the most common gold nanoparticle assemblies and morphologies.

Nanoparticles synthesized by the reduction of gold in aqueous phase tend to have quasi-sphere morphology as this shape presents the smallest surface area if compared to other morphologies. Typically, the suspension of spherical gold nanoparticles presents a ruby red color due to the scattering of light by the nanomaterial, but the increase in size, as well as a change in the environment surrounding the nanoparticles, may modify the optical properties of the colloid [16,18]. It is possible to synthesize gold nanoparticles in various forms, including nanospheres, nanorods, nanoshells, and nanoprisms, for a wide variety of applications. Figure 1 illustrates some of the morphologies that are featured by gold nanomaterials.

Another morphology of gold nanoparticle consists of a dielectric nucleus, i.e., made of silica, covered by a layer of gold, the so-called nanoshells. The width of the metallic layer determines the wavelength in which the surface plasmon resonance will be triggered from the ultraviolet region till near-infrared wavelengths where the tissue penetration is more intense [19]. By modifying the thickness of the metallic layer, the band corresponding to the surface plasmon resonance can be shifted towards different wavelengths including in the near infrared spectrum, which is a highlighted parameter for diagnostic purposes, especially cancer [20].

Gold nanorods have also gained special attention by researchers due to its easy synthesis and large surface available to interact with light per unit of volume if compared to other conformations. Because of their rod-like shape, it is possible to observe two surface plasmon resonance bands: the first band stems from the interaction of light with the electrons from the diameter width, and the other one, more red-shifted, stems from the interaction of light with electrons from the length width of the nanorods [21,22].

More recently, gold nanocages were developed as potential drug carriers. Their surface plasmon resonance peaks may also be tuned towards near-infrared wavelengths by adjusting the thickness of the walls and their porosity. Besides drug loading, these nanoparticles may carry other objects in their hollow interiors, such as smaller magnetic nanoparticles for different applications [23]. This complex structure can be synthesized by using silver nanocubes as templates in a galvanic replacement reaction in solution, as described further. Depending on the shape and, consequently, on the optical properties of the nanomaterials, they are useful as colorimetric sensors, catalysts, optical contrast agents for diagnostics in optical coherence tomography (OCT), agents for bioassay applications, or as hyperthermia-generating devices for cancer ablation, among many other applications [23].

A class of superconductors in nanometric scale have received the attention of researchers. The nitride compounds exhibit properties from ionic and covalent materials simultaneously, e.g., super hardness, high thermal and electrical conductivity, brittleness, magnetism, superconduction or superinsulation. Gold nitride, for instance, is a promising candidate for a plethora of applications, since jewelry, micro-engineering, catalysis and Raman spectrometry. These nitrides can be synthesized in different ways, with ease and good control of morphology and properties [24,25]. In a further section, we discuss the most important applications for the biomedical field.

#### 1.2.1. Methods of Synthesis: An Overview

There are several ways of synthesizing gold nanoparticles. The commonly used protocols are often categorized into top-down protocols (usually physical–chemical processes used to degrade a bulk material into smaller pieces, achieving the nanometric scale) or bottom-up protocols (the most abundant protocols for nanomaterials, where the syntheses of nanoparticles part from smaller precursors, such as metallic salts or molecular seeds that nucleate and form nanostructures) [26,27]. They can also be divided into physical, chemical and biological methods [28]. To a minor extent, supercritical fluid technology has also given some contributions in this regard. Together, those methods are applied to produce a great variety of nanoparticle morphologies, each one with specifically desired properties for a determined application [16]. Physical methods are based mostly on the energy transfer that occurs in a material when irradiated by ionizing or non-ionizing radiation, which may trigger the reduction reactions that lead to the nucleation of metallic particles. Those methods include photochemical processes [29,30], ionizing radiation [3,31], microwave radiation [32], among others.

Chemical routes are the most common and require strong and/or mild reducing agents, such as sodium borohydride (NaBH_4_) [33], hydrazine [34] and citrate [15] to initiate the synthetic process and promote nanoparticle nucleation. Porous supports are used to chemically synthesize nanoparticles with excellent control of size, since the morphology of the nanomaterials depends on the dimensions of the matrix pores, as demonstrated by Datta et al. [35]. These have been the most explored pathways for the synthesis of gold nanoparticles due to their ease of performance, high production yield and stability. Although NaBH_4_ and hydrazine are very efficient as reducing agents and have been used in several studies over decades, they are known to be biologically and environmentally toxic. That is why some phytochemicals have also been applied to the synthesis of gold nanoparticles in a green approach, including epigallocatechin [36], mangiferin [37], among others [38,39]. It is worth highlighting that such agents may also work as nanoparticles stabilizers to avoid precipitation or particle agglomeration.

Green approaches are becoming more common in nanomaterial applications. Microwave-induced plasma-in-liquid process (MWPLP) is a good example of green synthesis of nanoparticles as there is no need of using toxic reducing agents, and the energy consumed in the process is quite low. Similarly with radiolysis by gamma radiation or X-rays, MWPLP is based on the break of water molecules by microwaves, leading to the generation of reducing agents responsible for the nucleation of metallic particles [40]. Laser sources can be applied to the synthesis of gold nanoparticles as well. Correard et al. reported the synthesis of biocompatible gold nanoparticles using a Yb:KGW laser and the irradiation was performed until the solution became red. The nanoparticles presented excellent compatibility after the functionalization with polymers, and the synthesis led to a good morphologic control over the products [41].

The green nanotechnology concept has also gained significant power with the works of Katti and collaborators [36,37,38,39]. They have already used phytochemicals such as compounds from soybean extract. In this particular work, the low-molecular-weight proteins were able to initiate the nucleation (due to the action of amino acids such *L*-aspartic acid, *L*-lysine, *L*-tryptophan, *L*-tyrosine and *L*-arginine) but were not able to properly stabilize the generated nanoparticles, unlike the high-molecular-weight proteins, which demonstrated to be very efficient in the reduction of gold and stabilization of gold nanoparticles. The carbohydrates from the extract acted synergistically in the synthetic process, especially when carried out at higher temperatures [39].

There are antioxidants with polyphenolic carbon chain present in tea leaves, e.g., catechins (mainly epigallocatechin gallate (EGCG)) and theaflavins, which have also been investigated as reducing and capping agents in gold nanoparticles, and they demonstrated excellent initiating and stabilizing properties in the synthesis of 15–42 nm gold nanoparticles [36]. Cumins phytochemical constituents (for instance, aldehydes, alcohols, fats, and volatile oils), compounds bearing amino, thiol, hydroxyl, and carboxyl functional groups, were used as reducing agents, rapidly and efficiently generating gold nanoparticles that were further stabilized with gum arabic. All nanoparticles made through those green protocols demonstrated to be biocompatible and environmentally friendly [38]. Gold nanoparticles may also be synthesized using the microorganisms’ machinery, including bacteria and fungi, in the so-called biological methods. They also constitute an eco-friendly approach for the synthesis of such nanoparticles, as organic solvents are absent in this process [16,42,43].

In practical terms, one of the most common ways to synthesize gold nanoparticles, especially nanospheres, is by the reduction of AuCl_4_^−^ using citrate in an aqueous environment by the Turkevitch method [44]. In this case, citrate acts as both reducing agent and an anionic stabilizer, yielding nanospheres with approximately 15 nm in diameter according to the following reaction:

6AuCl_4_^−^ + C_6_H_8_O_7_ + 5H_2_O → 6CO_2_ + 24Cl^−^ + 6Au^0^ + 18H^+^ (1)

This protocol may be adjusted to produce nanoparticles up to 150 nm in diameter, either by modifying the concentration of citrate or by using gamma-radiation [44].

A seed-mediated method may also be applied for the synthesis of gold nanospheres with tunable diameters, according to the proportion between the precursor and the seeds. The small-diameter seeds are prepared by reducing AuCl_4_^−^ with a potent reducing agent, i.e., NaBH_4_ in the presence of citrate, used for further growth of the nanoparticles in the presence of a mild reducing agent such as ascorbic acid. Gold nanorods are also synthesized by this method [45].

Gold nanoshells—nanoparticles with a dielectric nucleus covered by a variable layer of gold—are typically prepared by direct deposition of gold onto colloidal silica spheres synthesized by the Stöber method [46] and with surfaces modified with a monolayer of amino-terminated silane. When gold nanoclusters with 1–2 nm are added to a suspension of those silica nanospheres, they are promptly attached to the amine groups. Then, more gold may be deposited on the surface via chemical reduction to cover the silica core, and the thickness of the metallic layer may be controlled appropriately, as above-mentioned [47].

Electrochemical methods or seed-mediated methods are used for the synthesis of gold nanorods. The first is conducted in a two-electrode cell with a gold layer being the anode and a platinum layer being the cathode, both immersed in an electrolyte solution containing a mixture of surfactants, such as hexadecyltrimethylammonium bromide (CTAB) and tetradodecylammonium bromide (TCAB). Small amounts of cyclohexane and acetone can be added to the electrolyte solution before the electrolysis, as acetone weakens the micellar network and cyclohexane facilitates the formation of elongated, rod-like CTAB micelles, necessary for the elongation of nanorods. During the electrolysis, bulk gold metal is converted from the anode into AuBr^−^ ions, which are driven by the electric current towards the cathode. The reduction occurs at the interface between the cathode and the electrolytic solution [48].

Gold nanocages consist of a complex structure whose synthesis is performed in a multistep process. Firstly, silver nanocubes (generated by reduction of silver in the presence of a polyol) undergo a replacement reaction with gold due to the higher standard reduction potential of the AuBr^−^/Au pair if compared to the Ag^+^/Ag pair. The reaction describing the galvanic replacement is the following:

3Ag(s) + AuCl_4_^−^(aq) → Au(s) + 3Ag^+^(aq) + 4Cl^−^(aq) (2)

Basically, at an early stage, the silver nanocubes bounded by {100} facets react with AuCl_4_^−^ to form small holes on a specific face, and as the replacement reaction goes on, the Au atoms resulting from the above reaction are epitaxially deposited onto the surface of the nanocube, generating a thin shell (sometimes the Ag atoms can form an alloyed Au-Ag shell). Then, the {111} facets at the corners of the resulting nanoboxes are dealloyed and further etched while more HAuCl_4_ solution is added, leading to the formation of a hole at each corner of the nanobox. The size of the holes and the thickness of the walls are controllable by adjusting the molar ratio of silver nanocubes and the HAuCl_4_ [49].

#### 1.2.2. Biomedical Applications

With the advent of nanotechnology, gold nanoparticles gained much attention due to the different optical and electrical properties obtained at the nanoscale, unlike the properties of gold in the macroscopic form. The combinations of these properties allow their application in the diagnosis and therapy of various diseases. In specific terms, the versatility in the ways of synthesizing and functionalizing gold nanoparticles opened a universe of research [28]. An updated summary of the current applications of gold nanoparticles is described in Table 1.

Some other uses of nanomaterials are colored nanoparticles as markers in immunoelectron microscopy in the field of cell biology or as immunochromatography dyes to detect influenza virus antigens and human chorionic gonadotrophin secreted in the urine during pregnancy [50,51]. Also, these materials may be used as drugs to treat several diseases, and within those, rheumatic ones are the most common (i.e., sodium aurothiomalate, aurothioglucose, and auranofin) [52,53]. Other uses address treatment agents for acquired immunodeficiency syndrome (AIDS), bronchial asthma, cancer and malaria [54], as well as sensors in colorimetric detection of heavy metals in blood [55] or of other chemical and biological molecules by gold nanoparticles [1], by chitosan/gold nanoparticle matrices [56] or by nanoporous gold wires synthesized in a green way [57].

In conventional chemotherapy, the agents are usually small molecules that present a broad and nonspecific distribution, and therefore may damage healthy tissues. Their action is also restricted by some types of cancer cell resistance mechanisms, e.g., high amounts of drug transporters expressed in the biological membrane, or expression of enzymes responsible for metabolizing such molecules. However, the use of gold nanoparticles may effectively assist in overcoming these problems as it is possible to anchor/functionalize these particles in/using several types of conjugates as demonstrated by Katti et al. [58]. In this research, the authors report the conjugation of gold nanoparticles with different phytochemicals and the respective assessment towards their tissue- and site-specificity and affinity.

The irradiation of nanoparticles using near-infrared (NIR) light produces heat, and thus may be applied in systems of heat-sensitive carriers for the controlled release of drugs, such as hydrogels and other “intelligent materials” [69]. The heat generated may act in synergy with the release of a chemotherapeutic drug from a heat-sensitive hydrogel formulation. The optical properties of the gold nanoparticles have been extensively explored and enabled their application as photosensitizers or as adjuvants in photothermic therapy, and as contrast and imaging agents [70,71].

Concerns about the use of gold nanoparticles for biomedical purposes are based on the pharmacokinetics of these compounds. Whether absorbed by intranasal, oral routes through the gastrointestinal tract, transdermal, or intravenous, they can penetrate capillaries regardless the type of tissue and can pass through epithelial membranes, thus affecting the physiology of cells [72,73].

#### 1.2.3. General Toxicity Aspects

Toxicity of nanoparticles has driven researchers in a continuous search for understanding possible toxicity mechanisms and pathways in humans, animals and in ecotoxicity models, including in vitro, ex vivo, in vivo approaches. The motivation beneath a better understanding of the nanotoxicity and related aspects relates to the fact that when such materials are brought to the nano-level, the toxicity properties are unlikely to be the same as the bulk material. The safety of nanomaterials for in vivo applications is doubtful because it depends on several parameters, such as size, surface chemistry, morphology, biological target, and charge [1,74,75]. Several lines of evidence have demonstrated no toxicity by nanomaterials, while several others demonstrated just the opposite. This divergence is due to a lack of homogeneity in the studies regarding the investigated parameters on the experiments [74]. At least a consensus exists on the idea that the damage occurring due to the presence of nanoparticles is mainly resultant of oxidative reactions, or by the binding of cationic nanoparticles to the anionic DNA, causing bends that lead to damage to the genetic material [1,74,76].

Chen et al., for instance, explored the lethality of nanoparticles and their toxicity associated with size, finding that particles ranging from 8–37 nm induce severe systemic adverse effects in an animal model, such as loss of appetite, weight loss, and altered skin color, in addition to a mortality rate of almost 100% for that specific range of size. However, the particles larger than 50–100 nm were found to be non-toxic, and no mortality was observed in animals [77]. This reduction of toxicity can be explained by the cellular semi-permeability that probably prevents or reduces the passage of larger molecules [78]. Other studies demonstrate that, in addition to the size cited above, the geometry and surface area also influence the toxicity of gold nanoparticles [74]. A study reported that positively charged spherical nanoparticles had a higher toxic effect than negatively charged nanoparticles [79]. In the same work, it was demonstrated that gold nanoparticles can cross the intestine, be absorbed into the bloodstream and distributed through heart, kidneys, lungs, liver, spleen and towards the central nervous system by crossing/overcoming the blood-brain barrier. Also, concerns towards possible teratogenic effects were highlighted as the nanoparticles were also capable of overcoming the placental barrier [80].

The toxicity of gold nanoparticles may also be influenced by the type of particle-coating, so many authors investigate and approach the toxicity taking into account aspects such as dose, size, shape, zeta potential and surface functionalization [81,82]. Furthermore, the protein corona (layer of proteins that adsorb on the nanoparticles in biological environments) exerts a significant influence on the cellular uptake, biodistribution and, consequently, toxicity [74]. The scientific challenges embroidered are related to the development of novel physical and chemical methods of synthesis and functionalization capable of conferring efficient binding, ease of purification, desirable clearance levels, biocompatibility, and reproducibility so that gold nanoparticles may be safely and efficiently applied in medicine [83,84]. Polyethylene-glycol (PEG), for instance, reduces the cellular uptake of nanomaterials, and therefore, is not a suitable polymer for nanomaterials. Other polymers (i.e., polyvinyl-pyrrolidone (PVP), poly (acrylic acid) (PAA), poly(allylamine hydrochloride) (PAH), and polyvinyl-alcohol (PVA)) and molecules (i.e., albumin and glutathione) can enhance the effectiveness and safety of nanomaterials in vivo [74].

Another important factor influencing the toxicity of nanoparticles is the time of exposure. Although the nanoparticles might seem safe for short-term uses, their accumulation in long-term exposures might lead to toxic effects, as demonstrated with gold nanoparticles by Gunduz et al. [85].

Bearing in mind that in vitro results may not be thoroughly representative of in vivo effects, and that trustful results arise from coordinated protocols performed by several authors, a homogeneity must be achieved by authors regarding the parameters used in their experiments, in order to provide trustworthy results and guarantee the safety for the use of nanomaterials in biomedical applications [74]. In vitro experiments to assess the toxicity of nanomaterials, for instance, might include proliferation assays (i.e., MTT cell viability assay), apoptosis investigation (i.e., Annexin-V assay, Comet assay, TUNEL assay), necrosis investigation (usually by evaluating membrane integrity by the uptake of Trypan Blue or Neutral Red dyes), and oxidative assays (investigating the content of reactive oxygen species or quantifying the antioxidant capacity of cells). Examples of in vivo experiments within this scope are usually related to the investigation of biodistribution, clearance, histopathology, and behavior in blood, as well as the assessment of the IC_50_ for each nanomaterial and, not least important, of the action mechanism behind the observed toxicity [86].

## 2. Radiation Technologies Applied to Gold Nanoparticles

### 2.1. Overview of the Radiolytic Synthesis of Gold Nanoparticles

Ionizing radiation refers to radiation emitted by electromagnetic waves or photons, such as gamma rays and X-ray, for instance, or from particle-like radiation, particularly in the case of electron beam radiation. Such particles or photons are known to hold the energy to break or induce chemical bonds, as well as to create electrically charged particles upon interaction with atoms or molecules apart from their source. The use of ionizing radiation has been highlighted as a useful tool for the development and synthesis of gold nanoparticles, among a plethora of other applications including medical device sterilization and polymer crosslinking, among others. In principle, the use of radiation for the synthesis of gold nanoparticles involves the solvent radiolysis, alternatively from the use of a chemical reductant, in which a solvent molecule is ionized and excited, thus generating a variety of reactive species that will then trigger the nanoparticle formation [14,87,88].

A highlighted contribution towards the fundamentals and principles of nucleation and mechanisms embroidered in the radiolytic formation of metallic atoms and clusters has been demonstrated by Belloni and colleagues at the end of the last century [88,89]. The fundamentals of radiation sciences categorize the radiation effects in two main streams—the direct and indirect effects. While direct effects are attributed to the energy transfer, the indirect ones occur via the interaction of the radicals or reactive species generated as a function of the direct effects over a solvent molecule, for instance. In most cases, especially regarding gold nanoparticle synthesis, the solvent is water. Water radiolysis is a well-defined and clarified process by now, capable of generating well-established highly reactive radicals, namely—OH^•^ (hydroxyl radical), H^•^ (hydrogen radical), e_aq_^−^ (solvated electron), HO_2_ (peroxide), and O_2_^•^ (superoxide), along with the following molecular species—H^+^, H_2_, O_2_, and H_2_O_2_ [90,91].

OH^•^, a highly oxidizing species, and the aqueous electron or so-called hydrated or solvated electron (e_aq_^–^), a very potent reductant agent [92], are the most important byproducts, primary radicals originated from water radiolysis, that play a major role when it comes to the synthesis of gold nanoparticles. The e_aq_^–^ and the H^•^ are responsible for the nucleation process, which mediates the reduction of Au^+3^, while the presence of OH^•^, if not adequately scavenged, may lead to an opposite effect due to the highly oxidizing properties which may counterbalance the reductions [2,89]. If properly scavenged, they may lead to secondary radicals of reductant nature, which will help the nucleation properties, e.g., alcohol-derived radicals. The generation of such species is directly related to the type of radiation and the LET, the reaction media, and the atmosphere in which irradiation is carried out.

At a second stage, after nucleation, the formation of a free cluster of controlled size in aqueous media is not stable, as aggregations process tends to continue until high nuclearity values are achieved and thus leading to precipitation. Therefore, a proper stabilizer, e.g., a ligand, capping agents or surfactants, is essential to inhibit or block the aggregation process, so coalescence ceases whether by electrostatic repulsion or steric hindrance [88].

#### 2.1.1. Radiation Sources

The most commonly used ionizing radiation types for the radiolytic synthesis corresponds to UV light, gamma-ray, X-ray and electron beam [3]. Although UV light is considered ionizing at specific UV wavelengths, this review rather focuses on the uses of gamma, X-ray and electron beam radiation. Although the solvent radiolysis occurs when using gamma, electron beam or X-ray, the LET affects the spatial distribution of the reactive species produced, influencing the nucleation and growth of nanoparticles. Low LET radiation, i.e., γ-rays, leads to a higher yield of reducing species per energy unit deposited along their path, thus generating smaller particles with narrow size distribution [3].

Gamma irradiators are typically applied for the sterilization of medical devices and equipment, polymer processing and radiation treatment of food products [93]. The technology is based on cobalt-60 or cesium-137 as radioactive sources and therefore not on-off switchable. However, Cesium-137 use is restricted to small, self-contained dry storage irradiators [94]. Penetration is a crucial feature of this technology. Gamma rays consist of photons capable of penetrating up to 300 mm into materials (depending on the density) due to their absence of mass. In practice, this allows the processing of products on a large scale and in larger quantities at once [94]. Within this context, the replacement of gamma irradiators for high dense products seems unlikely. However, proper handling and replacement of the radioactive sources, decommissioning and other related activities are mandatory, apart from demanding equipped structures [94].

X-rays are another example of low LET radiation with more accessible radiation sources that are on/off switchable and have attracted attention over the recent years. Similar to gamma radiation, they have a strong penetration power as they consist of high-energy photons. Particularly powerful X-ray (bremsstrahlung) radiation sources are of highlighted interest [94].

On the other hand, gamma and X-rays may deliver a considerably lower radiation dose rate (four to five orders of magnitude) if compared to electron beam technology, although X-ray may provide higher dose rate if compared to gamma. Consequently, the product throughput is inferior in contrast to electron beam [94]. Besides, X-ray devices enable the in-situ characterization during the synthesis of nanomaterials by the study of metal ion reduction, nucleation and growth in real time if coupled with small angle X-ray scattering (SAXS) and UV-Vis techniques. Higgins et al. reported the synthesis of gold nanoparticles supported on titania (TiO_2_) using X-ray radiolysis and confirmed the efficacy of the method [95].

Electron beams have also been extensively explored for the synthesis of gold nanoparticles [96,97,98]. The currently available industrial electron accelerators are usually categorized according to their energy, in which low-energy accelerators are related to machines with beam power ranging from 300–350 kW and energy around 0.15–0.5 MeV, while intermediate-energy accelerators are placed within those with beam power around 300–350 kW and energy 0.5–5 MeV. As for high-energy accelerators, the energy is around 5–10 MeV with energies up to 100 kW. For instance, the expected penetration of a high energy accelerator of 10 MeV is around ~38 mm, depending on the density of the material [94,99]. The technology holds high throughput and offers as an advantage a recognized operational safety and power [94,99].

X-ray and electron beam alike regarding no need for a radionuclide, or a radioisotope, and thus being recognized as a switchable radiation source, as above-mentioned. However, electron beam accelerators abound worldwide, unlike X-rays, which are an emerging technology from an industrial perspective, but are outnumbered when compared to gamma or electron beam facilities. Additional favorable aspects related to electron beam technologies over gamma, for instance, rely on the ability to be adjusted for in-line processing, and better acceptability by the general public, apart from the absence of decommissioning, transport and storage steps inherent to radioactive sources [94]. Electron beams also allow the delivery of an intense radiation dose rate, thus processing may be performed in a reduced time-scale. Some accelerators also enable the possibility to control the dose rate to some extent. Limitations of electron beam irradiation over gamma are related to the penetration, which strongly depends upon the energy of the accelerators, which may vary from a few hundred keV to 10 MeV as aforementioned. Some of the radiolytic protocols for the synthesis of gold nanoparticles are detailed in Table 2.

#### 2.1.2. The Radiolytic Mechanism of Gold Nanoparticle Formation

The synthesis of gold nanoparticles via radiation, from a general perspective, is direct and controllable, since changing a few parameters may lead to tunable particle size and shapes. However, a series of complex reactions are involved in the radiolytic synthesis of gold nanoparticles. At initial stages, ionizing radiation produces high amounts of H^•^ atoms and hydrated electrons (e_aq_^−^) via the radiolysis of aqueous solvents, and these species present strong negative potentials of −2.3 V_NHE_ and −2.87 V_NHE_, respectively [3]. Thus, as strong reducing agents, they readily reduce metal cations into zero-valent metal nanoclusters and nanoparticles, according to the following equations:

H_2_O + radiation → e_aq_^−^, H_3_O^+^, H^•^, H_2_, OH^•^, H_2_O_2_ (3)

Au^3+^ + 3e_aq_*^−^* → Au^0^ (4)

Au^3+^ + 3H^•^ → Au^0^ + 3H^+^ (5)

The quantity and the size of the newly-formed metallic nuclei are controllable by simply adjusting the irradiation parameters, especially the dose and the dose rate. One necessary step in this process is the addition of a hydroxyl radical OH^•^ scavenger since this species is also generated during radiolysis and presents a strong positive potential of +2.8 V_NHE_. Therefore it can oxidize the metal ions or the newly-formed atoms into a higher oxidation state [3,110]. Examples of hydroxyl radical scavengers are primary or secondary alcohols, acetone, and formate ions. The most commonly used one is isopropanol, which scavenges hydrogen and hydroxyl radicals generating a secondary radical with strong negative potential. This new radical can now further contribute to the reduction of gold ions into their zero-valent form, according to the equations [111]:

OH^•^ + CH_3_CHOHCH_3_ → H_2_O + H_3_CC^•^OHCH_3_ (6)

H^•^ + CH_3_CHOHCH_3_ → H_2_ + H_3_CC^•^OHCH_3_ (7)

Au^3+^ + 3H_3_CC^•^OHCH_3_ → 3CH_3_COCH_3_ +Au^0^ + H^+^ (8)

The zero-valent atoms may then act as nucleation centers and enable further coalescence. It is important to mention that the nucleation and coalescence (described in the equations below) are favored in these systems because the metal atom-atom and metal atom-ion binding energies are stronger than the atom-solvent or atom-ligand binding energies [110,112].

Au^0^ + Au^0^ → Au_2_ (9)

Au^0^ + Au^3+^ → Au_2_^3+^ (10)

**Figure 2:**
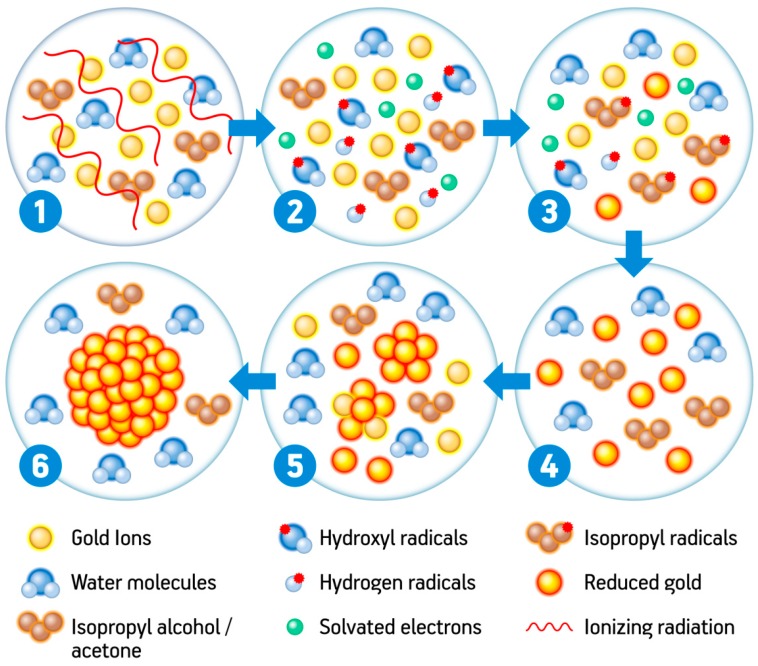
Representative scheme of the synthesis of gold nanoparticles using high energy radiation.

The nucleation goes on until stabilization of the system. Due to the large surface area, all nanomaterials present intense surface energy and are, therefore, thermodynamically unstable or metastable. The nanoparticles in a colloid are attracted to one another by van der Waals interactions, so in the absence of a counteracting force, aggregation and destabilization of the colloidal system are likely to take place [3,110]. A summary of the radiolytic synthesis of gold nanoparticles is shown in Figure 2.

#### 2.1.3. Capping and Stabilizing Agents

As briefly mentioned before, one of the main characteristics of nanomaterials relates to the presence of high surface energy derived from their large surface area. Therefore they become unstable or metastable thermodynamically. Besides, in a colloidal system, nanoparticles tend to aggregate due to the mutual attraction caused by van der Waals forces, which destabilizes the system. Therefore, several strategies have been developed hitherto aiming the passivation of the nanoparticles, in other words, to overcome the large surface energy and to prevent aggregation, based either on electrostatic or on steric stabilization [3].

On one hand, electrostatic stabilization consists of the effect of the repulsive electrostatic force that the nanoparticles go through when there is a double layer of electric charges around them. According to the Derjaguin–Landau–Verwey–Overbeek theory, the total energy potential of the interaction between two colloidal particles is the sum attractive forces (van der Waals) and repulsive forces (coming from the double layer of electric charges). If the total energy potential is higher than the kinetic energy of the particle motion, the particle is considered stable [113]. On the other hand, the stability or instability of particles cannot only be described by the electrostatic stabilization, but the surface energy must also be taken into consideration, as mentioned above. Metal surfaces tend to have a surface energy ranging from 1000 to 2000 mJ m^−2^, values much higher than the ones observed for other organic and inorganic materials with energies much lower than 500 mJ m^−2^, and this high surface energy along with additional attractive dipole–dipole interactions contribute to the instability of these nanomaterials [113].

Steric stabilization consists of the repulsion between molecules or ions adsorbed on neighboring particles. The larger the adsorbed molecules, the more effective they are in stabilizing the nanoparticles due to the geometric constraints they cause. The spatial conformation of the molecules is important as well, since it was demonstrated that elongated or conical conformations lead to an improved stabilization. If the size of the nanoparticle is shorter than the length of the stabilizer, as in the case of long-chain polymers, an encapsulation process may occur, and the particle is thus passivated. Furthermore, molecules presenting chemical groups with a free electron pair, such as trivalent phosphorus, divalent sulfur, trivalent nitrogen moieties, or molecules with π-electrons that are as aromatic compounds, tend to adsorb very strongly onto metal surfaces [111] and are good passivating agents.

A plethora of colloidal metal stabilizers or passivating agents are available, and the choice among them depends on the type of metal, the protocol of synthesis, the application, and the desired nanoparticle characteristics. Passivating agents capable of encapsulating the newly-formed nanocrystals, preventing them from aggregating by restricting the access of reactants to the crystals (steric hindrance by covalent binding to the nanomaterials) are also capable of providing the nanomaterials with specific characteristics, as inertness to the nonspecific adsorption of biomolecules and stress responsiveness as an intelligent carrier, making them prone to be homing agents for targeting particular tissues. Inorganic compounds, especially metal oxides, are also applied as nanoparticle stabilizers. They were originally used as catalyst supports, i.e., Al_2_O_3_ supported on Ni nanocluster, whose amphoteric character plays an important role on the fixation of metal ions [3].

Passivating chemicals can decrease the polydispersity of nanoparticles as well, by chemically stabilizing the nanocrystals, i.e., ligands bearing thiol groups; and to alter the resultant crystal shape by using variable amounts of capping agents such as polymers or small molecules bearing surface-active functional groups, i.e., polyvinylpyrrolidone (PVP) [114].

The decrease of polydispersity with the use of stabilizing agents is a consequence of a significant influence on the size and morphology of the nanoparticles by those compounds. In the case of PVP, for instance, the size of the product decreases with the augment of polymer concentration in the synthesis medium. This might occur because higher amounts of polymer in the solution affect the motion of the reduced metal, thus limiting the aggregation of colloids into bigger particles [114]. PVP was also used as a capping agent for the synthesis of other metallic nanoparticles using thermal treatment [115]. The same effect can be observed with primary amines during the synthesis of gold nanoparticles, but since these compounds are the reducing and the passivating agents simultaneously, the influence on the particle size is due to the rapid thermal decomposition of the gold-amine complex in water at high temperatures and the subsequent strong protection of the gold nanoparticles by the amines, preventing further aggregation [116]. Polyelectrolytes such as linear polyethyleneimine (LPEI) can also be used as reducing and passivating agents (the passivation occurring due to both steric and electrostatic stabilization), and a similar concentration-derived effect can be observed [117].

The polymer chain size also plays a role in tuning the characteristics of the final particles, as demonstrated by Luo et al. (2005) using polyethylene glycol (PEG) as the reducing and passivating agent of silver nanoparticles. The authors found that a higher silver reduction rate was observed with heavier PEG chains, and this influenced the size, shape, and polydispersity of the final product significantly [118]. For the growth of anisotropic gold nanoparticles, cetyltrimethylammonium bromide (CTAB), poly (ethyleneimine) (PEI), PVP, citrate and small amine molecules are commonly used as passivating agents. On the other hand, thiol compounds are more used for isotropic nanoparticles, and are not indicated for the growth of anisotropic crystals [114].

It was described in previous sections that some natural compounds can act as both reducing and stabilizing agents in the synthesis of gold nanoparticles. Polyphenolic compounds such as EGCG, high-molecular-weight proteins from soy extracts, and cumin constituents, are efficient, biocompatible and environmentally friendly substances that can be freely used to initiate the nucleation of gold nanoparticles and to prevent aggregation, sometimes with the aid of other natural product such as Gum Arabic [36,38,39,119].

When it comes to radiolytic synthesis, however, polymers such as PVP and polyvinyl-alcohol (PVA) are the most common stabilizers. Polyvinylpyrrolidone associates with metal nanoparticles by the functional groups C=O and N, which have lone pairs of electrons that help the stabilization of the nanomaterials at their surfaces by covalent binding, whereas the long polymer chain exerts a steric hindrance that restricts the interaction with other nanoparticles, thus preventing aggregation [120]. PVA chains, on the other hand, avoid nanoparticle aggregation due to the inhibition of metal hydroxide clusters by hydrolysis of metal ions [121].

In conclusion, the choice of passivating agents must not be random, but rather, should take into consideration the morphology and the desired application of the nanomaterial, and might then require adjustments in the synthetic protocol according to the nature of the compounds.

#### 2.1.4. Nanoparticle Tuning

Several technologies abound for the synthesis of gold nanoparticles with advantages and setbacks. However, a considerable need for a method that allows the design of nanoparticles in line with the requirements for biomedical applications, especially concerning overcoming biological barriers and other biopharmaceutical aspects with negligible or reduced toxicity has been highlighted. As for the radiolytic synthesis, several conditions and parameters may be adjusted towards controlling the nucleation process, which directly influences nanoparticle characteristics. Among those, solvent, radiation dose and dose rate, pH, temperature and precursor concentration play a remarkable role [110].

Regarding the solvent, substantial evidence has been provided towards the influence of the atom-solvent interaction, as most of the properties, including optical absorption spectra, are solvent-dependent. Polarity seems to play an important role, as dielectric constant changes [122,123]. Also, the presence of reducing agents in the solvent plays the most crucial role on the reduction velocity, affecting particle size and distribution (the faster the reaction, the smaller the particles, but the wider the size distribution). As an example, hydrated electrons produced by water radiolysis are stronger reducing agents than 2-propyl radicals, so the latter is more suitable for achieving a narrower size distribution [3]. Within this context, a proper selection of a solvent allows the tuning of nanoparticle characteristics during the radiolysis process.

The pH seems to influence the size obtained for the nanoparticles as well since the surface plasmon resonance band from the nanoparticles tend to red-shift according to a pH increase, accompanied by a decrease in stability and consequent higher tendency to agglomeration [3]. Also, the pH and temperature change the G-values of the generation of the reactive species [90], and in that sense, they comprise relevant tools for tuning nanoparticle size as well.

**Figure 3:**
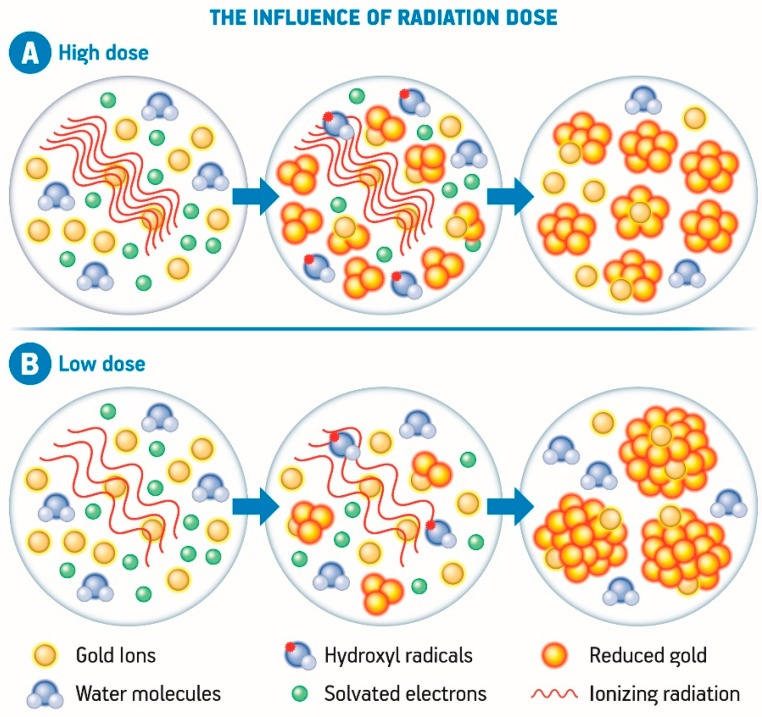
Representative scheme of the influence of high (**A**) and low radiation dose (**B**) over the nucleation and growth of gold nanoparticles generated by the radiolytic synthesis using high energy radiation.

The radiation dose is another factor that exerts influence on the growth rate and the size of nanomaterials, and this is more evident in the case of bimetallic nanoparticles. It has been demonstrated that at low radiation doses, the consequent low reduction rate leads to a smaller quantity of metal nuclei than the number of metal ions. Consequently, the exceeding metal ions may ionize bimetallic nanoparticles leading to the formation of bigger particles by the reduction and aggregation processes, as elucidated in Figure 3. On the other hand, if a high radiation dose is applied, most of the metal ions are consumed during the nucleation process leading to a lower concentration of unreduced metal ions compared to the nuclei. The nanoparticles are, therefore, smaller in size when a higher radiation dose is applied (Figure 3) [3,110,122,123].

**Figure 4:**
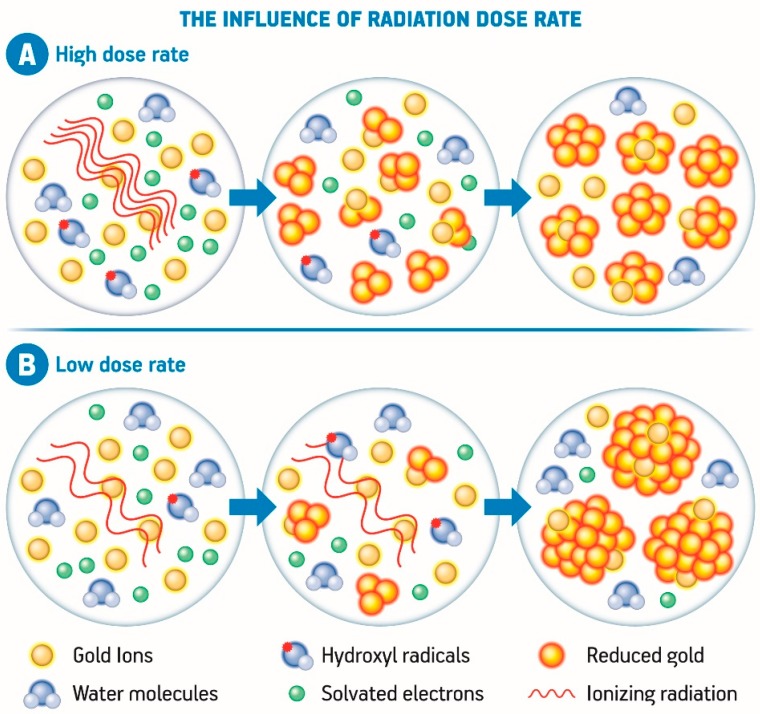
Representative scheme of the influence of high dose rate (**A**) and low dose rate (**B**) over the nucleation and growth of gold nanoparticles generated by the radiolytic synthesis using high energy radiation (Adapted from [108,109]).

Another aspect to be considered is the radiation dose rate. At a high dose rate the radicals are produced in a short time scale which leads to coalescence of the atoms separately formed, whereas at a low dose rate, the production rate of the radicals is slower than the coalescence and dimerization processes. In practical terms, low dose rates often generate nanoparticles with higher size as well as high dose rates are associated to lower nanoparticle size when compared to one another [123,124], as demonstrated in Figure 4. Another case in which larger clusters can be formed is when an electron donor compound is added to the system. In this case, the reduction potential is usually not sufficient to form new nuclei, so the reduction of ions onto the previously formed nuclei act as seeds, enabling the increase of the final clusters [110,123,124].

The final size of metal nanoparticles also relies on the concentration of the precursor metal, since the higher the precursor concentration is, the larger the nanoparticles become. There are three main reasons for this to occur. One of them is the fact that the rate of ion association that forms larger particles increases with higher concentrations of the precursor ions. Besides, the viscosity of the solution changes as the polymer/ion ratio is altered, consequently changing the speed of particles in the solution. Finally, as the surface energy and further agglomeration of nanoparticles are reduced by the adsorption of polymers on the surface of metal nanoparticles, if the ion concentration increases, the capping performance of the polymer onto the surface of nanoparticles is reduced leading to the formation of larger nanoparticles [3,110].

#### 2.1.5. Advantages of the Radiolytic Synthesis

Many benefits may be outlined when radiation is applied towards the development of advanced and optimized methods of gold nanoparticles synthesis when compared to other approaches or conventional technologies, including superior yields of monosized and highly monodispersed metallic clusters. Other aspects and possibilities of the radiolytic synthesis include the performance of the experiments under mild conditions of temperature and pressure, including the lack of chemical reducing agents, with high reproducibility. Experimental protocols lead to the synthesis of gold nanoparticles with high degrees of purity, achieved apart from the presence of chemical reducing agents, and confer the possibility of controlling particle size and structure [3,111].

Another useful aspect of this technology is the flexibility in terms of radiation sources, as it can be performed by using gamma, e-beam or X-rays or even UV-light sources, without impairment of the final product nor need for formulation changes, apart from adjusting the radiation source.

The possibility to combine sterilization and nanoparticle formation simultaneously in a single process is a unique advantage achievable by using ionizing radiation. This process occurs via simultaneous effect over pathogenic or contaminating microorganisms and the material or ion which leads to the nanoparticle formation simultaneously, resulting in a much quicker, simple and cost-effective process if compared to other routes for the synthesis of gold nanoparticles and other materials. No residuals nor relevant damage to the product is expected if the irradiation process is properly performed and adjusted. This advantage is even more pertinent if the developed system corresponds to the final product to be commercialized, since radiation may also be performed inside the final packaging, thus avoiding further contamination and excessive handling.

## 3. Final Remarks

There was a significant contribution from the radiolysis of solutions containing metal ions to the knowledge concerning the exceptional properties presented by free atoms and oligomer clusters in suspension, as well as about their nuclearity-dependent redox potentials. This particular approach enabled the synthesis of various nanomaterials with applications from catalysis in solution to nanodevices for cancer ablation, among several other applications, which, distinctively from a majority of the available methods, allows the reaction to occur in the absence of chemical reducing agents or additional toxic compounds. The lack of such compounds during manufacturing contributes to the low toxicity of the final product and reduces the need for associated washing or removal stages.

The current trends in the nanotechnology field or the so-called nano-era is all towards optimizing the reaction conditions to give more monodisperse nanoparticles, as well as confer the decoration or proper assembly of those nanoparticles according to the desired purpose to optimize the material for final applications, especially concerning biotechnological and biomedical fields. In that sense, much attention has been given to the functionalization of gold nanoparticles for biomedical applications, or to the enhancement of radiotherapy parameters by using metallic nanoparticles administered to the tumors with enhanced selectivity and biological affinity.

Within this context, techniques capable of providing gold nanoparticles of controllable size with improved biological compatibility and negligible-toxicity are significantly relevant. With the use of the radiolytic synthesis, nanoparticles synthesis and functionalization may be carried out in a single step, followed by simultaneous irradiation which highlights the advantages of the technique to overcome the issues faced by conventional pathways for the synthesis of gold nanoparticles. However, there are several mechanistic points to be elucidated, and improvements to be done in the process of those nanomaterials on a synthesis and an application-based approach.

## Figures and Tables

**Table 1:** An updated review of the applications of gold nanoparticles (AuNP).

Method of Synthesis	Nanoparticle Properties	Applications	Ref/Year
Citrate reduction of chloroauric acid	Size of 35 nm (TEM) and SPR peak at 529 nm	Molecular biosensor techniques for the diagnosis of cancer	El-Sayed et al., 2005 [59]
Citrate-stabilized AuNP followed by grafting of polymers onto the NP	Size of 14 nm and stable for at least 3 months (25 °C)	Photothermal therapy and chemotherapy	Song et al., 2012 [60]
Radioactive gold (^198^Au) NP produced using epigallocatechin	Size of 80 nm, SPR at 535 nm	Nanotherapeutic agent in oncology	Shukla et al., 2012 [61]
Gold nanorods prepared by the seed-mediated method and encapsulated by silica and other compounds	Sizes from 5 nm to over 25 nm	Simultaneous multimodal tumor detection and photodynamic therapy	Zang et al., 2013 [62]
Alpha-tocopheryl succinate conjugated multifunctional dendrimer-entrapped AuNP using ice-cold NaBH_4_ solution	Water-dispersible 3.3 nm (core size) AuNP, SPR peak at 570 nm	Platform for targeted cancer imaging and therapyf	Zhu et al., 2014 [63]
Branched gold nanoshells produced by a seeded-growth method lacking surfactant	Size around 135 ± 25 nm (DLS), SPR peak at 490 nm	Simultaneous cancer therapy	Topete et al., 2014 [64]
Citrate-capped, cysteamine-capped, and naked AuNPs	Size (DLS) from approximately 17 to 100 nm	Antibacterial agent	Tao et al., 2015 [65]
Micelles upon transferrin conjugation prepared by the solvent casting method	Sizes from 16.4 ± 0.39 nm to 20.3 ± 0.68 nm	Agent for cancer imaging, therapy, and theranostics	Muthu et al., 2015 [66]
Hybrid nanocomposite synthesized by Au deposition onto docetaxel-loaded poly (lactide-co-glycolide)	Size around 180 nm, SPR peak at 520 nm	Tumor-targeted chemo-photothermal therapy	Hao et al., 2015 [67]
Au^3+^ is partially reduced to Au^+^ by the subsequent addition of a thiol with simultaneous formation of Au(I) thiolate oligomers in an organic solvent	Size around 12 nm with emission from blue to NIR	Optical imaging and theranostics	Cantelli et al., 2016 [68]

SPR—Surface plasmon resonance; NIR—Near Infra-Red; Au: Gold.

**Table 2:** An updated review of gold nanoparticles synthesized by ionizing radiation reported in the literature.

Radiolytic Approach	Nanoparticles Properties	Applications	Ref/Year
Gamma irradiation (^60^Co) at 10 kGy and dose rate of 19.6 Gy min^−1^ with a quaternary ammonium-based ionic liquid	The presence of QAIL led to smaller and more stable nanoparticles with a size of 12 nm (TEM), 10.6 nm (X-ray diffraction), and 34 nm (DLS)	These nanoparticles can be used as catalysts and in electrochemistry	Chen et al., 2005 [100]
Synchrotron X-ray irradiation for 90 s (2.5 GeV and 150 mA) using NaHCO_3_	Particle size ranged from 15 to 20 nm and sizes > 1 µm at higher NaHCO_3_ content	Promising applications as drug carriers	Yang et al., 2006 [101]
Gamma irradiation (^60^Co) of aniline carbon nanotubes (3 kGy) containing CTAB and HAuCl_4_ in N_2_ atm	AuNP of 5 nm decorated onto the surface of single-wall carbon polyaniline coated nanotubes	Sensors, electrocatalysts and in microelectronics	Lee et al., 2007 [102]
Gamma irradiation for 3 h (^137^Cs) dose rate of 1.8 kGy h^−1^ or UV (15 min, Hg lamp, 200 W, 235 nm, 30 cm)	Sizes of 5.9 ± 1.7 nm upon UV and 2.9 ± 0.7 nm after gamma irradiation	Biomedical, chemical, and electronic purposes	Meyre et al., 2008 [103]
Gamma irradiation (^60^Co) using 2.5 to 10 kGy, dose rate of 5.4Gy s^−1^ containing BSA	Sizes of 7.5 nm (2.5 kGy), 2.7 nm (5 kGy), and 2.3 nm (10 kGy) with a spherical shape	Pharmaceutical and biomedical applications	Akhavan et al., 2010 [104]
Gamma irradiation (^60^Co, dose rate of 3.4 kGy h^−1^) of HAuCl_4_ solution containing CTAB	The authors obtained gold nanorods with an average aspect ratio of 3.0	Potential applications as chemical sensors	Biswal et al., 2010 [105]
E-beam irradiation (doses of 5 to 50 kGy, dose rate of 15 kGy s^−1^) compared to gamma (^60^Co) at doses of 7.8 kGy to 23.4 kGy, dose rate of 1.1 kGy h^−1^ containing chitosan	Sizes of 4.2 nm stabilized with chitosan (γ) and 27 nm (5 kGy), 12 nm (10 kGy) and 7 nm (15 kGy) by electron beam	Biomedical and technological applications	Vo et al., 2014 [106]
Gamma irradiation (^60^Co) doses of 1, 10 and 30 kGy, dose rate of 8 kGy h^−1^, containing citrate in N_2_ or air	Size of 10 nm (in air), twice as much as the nanoparticles synthesized in a nitrogen atmosphere	Targeting agents for cancer upon the surface modification	Hanžić et al., 2015 [107]
Gamma irradiation (^60^Co) at dose rate of 1.5 Gy s^−1^ (150 rad s^−1^) at 30 °C	Size ranged from 2 to 22 nm confirmed by the broadness of its SPR peak	γ-irradiation based strategy for metal NPs preparation	Abdelghany et al., 2017 [108]
X-ray irradiation up to 35 Gy, dose rate of 15.6 Gy min^−1^ in presence of CTAB and AA	Sizes (DLS) of 121.1 ± 20.7 nm (5 Gy) to 57.3 ± 3.97 nm (35 Gy)	Measurements of ionizing radiation in diverse areas	Akar et al., 2018 [109]

Atm: Atmosphere; AA Ascorbic Acid; Co: Cobalt; Cs: Cesium; CTAB: Cetrimonium Bromide.
